# Impact of Previous Virological Treatment Failures and Adherence on the Outcome of Antiretroviral Therapy in 2007

**DOI:** 10.1371/journal.pone.0008275

**Published:** 2009-12-14

**Authors:** Marie Ballif, Bruno Ledergerber, Manuel Battegay, Matthias Cavassini, Enos Bernasconi, Patrick Schmid, Bernard Hirschel, Hansjakob Furrer, Martin Rickenbach, Milos Opravil, Rainer Weber, the Swiss HIV Cohort Study

**Affiliations:** 1 Division of Infectious Diseases and Hospital Epidemiology, University Hospital Zurich, University of Zurich, Zurich, Switzerland; 2 Division of Infectious Diseases and Hospital Epidemiology, University Hospital, Basel, Switzerland; 3 Division of Infectious Diseases and Hospital Epidemiology, University Hospital, Lausanne, Switzerland; 4 Division of Infectious Diseases and Hospital Epidemiology, Cantonal Hospital, Lugano, Switzerland; 5 Division of Infectious Diseases and Hospital Epidemiology, Cantonal Hospital, St. Gallen, Switzerland; 6 Division of Infectious Diseases and Hospital Epidemiology, University Hospital, Geneva, Switzerland; 7 Division of Infectious Diseases, University Hospital Bern, and University of Bern, Bern, Switzerland; 8 Swiss HIV Cohort Study, Centre Hospitalier Universitaire Vaudois, Lausanne, Switzerland; Institut National de la Santé et de la Recherche Médicale, France

## Abstract

**Background:**

Combination antiretroviral treatment (cART) has been very successful, especially among selected patients in clinical trials. The aim of this study was to describe outcomes of cART on the population level in a large national cohort.

**Methods:**

Characteristics of participants of the Swiss HIV Cohort Study on stable cART at two semiannual visits in 2007 were analyzed with respect to era of treatment initiation, number of previous virologically failed regimens and self reported adherence. Starting ART in the mono/dual era before HIV-1 RNA assays became available was counted as one failed regimen. Logistic regression was used to identify risk factors for virological failure between the two consecutive visits.

**Results:**

Of 4541 patients 31.2% and 68.8% had initiated therapy in the mono/dual and cART era, respectively, and been on treatment for a median of 11.7 vs. 5.7 years. At visit 1 in 2007, the mean number of previous failed regimens was 3.2 vs. 0.5 and the viral load was undetectable (<50 copies/ml) in 84.6% vs. 89.1% of the participants, respectively. Adjusted odds ratios of a detectable viral load at visit 2 for participants from the mono/dual era with a history of 2 and 3, 4, >4 previous failures compared to 1 were 0.9 (95% CI 0.4–1.7), 0.8 (0.4–1.6), 1.6 (0.8–3.2), 3.3 (1.7–6.6) respectively, and 2.3 (1.1–4.8) for >2 missed cART doses during the last month, compared to perfect adherence. From the cART era, odds ratios with a history of 1, 2 and >2 previous failures compared to none were 1.8 (95% CI 1.3–2.5), 2.8 (1.7–4.5) and 7.8 (4.5–13.5), respectively, and 2.8 (1.6–4.8) for >2 missed cART doses during the last month, compared to perfect adherence.

**Conclusions:**

A higher number of previous virologically failed regimens, and imperfect adherence to therapy were independent predictors of imminent virological failure.

## Introduction

Combination antiretroviral therapy (cART) has dramatically reduced morbidity and mortality of HIV-infected persons with access to care. Nevertheless, therapeutic failure still remains substantial, in particular due to late initiation, interruption or refusal of cART, incomplete adherence to therapy, medication toxicities, antiretroviral drug resistance, hepatitis virus co-infections, consumption of alcohol, illicit drug use, or depression.

The potency of ART regimens has continuously improved but virological outcome is still not optimal. A large pan-European collaboration recently published on responses to cART across age groups and observed the best virological outcomes for older patients with up to 80% having reached viral suppression to <50 copies/ml by 3 years after initiating cART [1]. Recent randomized controlled trials of cART in treatment-naive persons showed viral suppression to <50 copies/ml in up to 85% of study participants at 48 weeks in intent-to-treat analyses [2]–[4]. Fortunately, significant progress has also been made among treatment-experienced persons in whom rates of complete viral suppression as high as 65% were reported at 48 weeks if new drug classes were applied [5]. However, randomized trials are not designed to generate long-term results and, because of generally very selected, well motivated and closely monitored patient groups, results from clinical trials are not readily applicable to the general patient population.

### Objectives

The aims of this study were to analyze determinants of virological failure in all HIV-infected persons on cART prospectively followed in a large national cohort study during 2007. Further, we wanted to describe the frequency of treatment modifications and discontinuations, as well as the clinical course. We were especially interested in the history of previous treatment failures and adherence as predictors for imminent virological failure.

## Methods

### Participants

We selected participants of the Swiss HIV Cohort Study (SHCS) who were enrolled prior to 2007, were on uninterrupted cART for ≥3 months at their first cohort visit in 2007 (visit 1); and had one additional semiannual follow-up visit prior to June 30, 2008 (visit 2). Patients were categorized into two groups according to the era of antiretroviral treatment initiation, i.e. mono/dual drug therapy vs. cART era. We excluded patients who started with drug combinations not clearly attributable to mono/dual drug regimens or cART, unavailable CD4 cell counts, HIV-1 RNA or adherence data within 6 months prior to visit 1 or at visit 2.

### Description of Procedures or Investigations Undertaken

Patients were assigned to the mono/dual drug treatment era if their initial regimen consisted of ≤2 nucleoside reverse-transcriptase inhibitors (NRTI), or three NRTI's without abacavir prior to 1999. Patients starting with ≥3 drugs including a protease inhibitor (PI) or a non-nucleoside reverse-transcriptase inhibitor (NNRTI) or abacavir in addition to two other NRTI were assigned to the cART era.

Previous regimens were defined as virologically failing from the date of the first available HIV-1 RNA record onward if ≥2 consecutive HIV-1 RNA measurements were >400 copies/ml, or ≥1 measurement was >1000 copies/ml, while the patient was on the same regimen for ≥3 months. In accordance with a UK-CHIC study [6], any mono/dual drug regimen taken before cART was counted as one additional failed regimen, because they were generally not virologically suppressive and HIV-1 RNA was not routinely measured at that time. Each individual treatment regimen was counted only once as virologically failed, even if repeatedly used.

Self-reported adherence was classified according to the number of missed doses within four weeks prior to a cohort visit (0, 1, 2, or >2 missed doses) as described previously [7].

Hepatitis B virus (HBV) infection was considered active if HBs antigen, HBe antigen or HBV DNA were positive. Hepatitis C virus (HCV) infection was considered active if anti-HCV antibodies and HCV RNA were positive; and inactive if HCV serology was positive and HCV RNA negative.

Virological failure was defined as having a HIV-1 RNA ≥50 copies/ml at visit 2. Treatment discontinuation was defined as ≥15 days off cART between the two visits. Treatment modification was recorded if at least one drug of a regimen was modified between the two visits. Treatment interruptions lasting less than 15 days were considered as treatment modifications. We also considered new AIDS-defining clinical events or death occurring between the two visits.

### Ethics

The SHCS is a prospective cohort study, established in 1988, with semi-annual follow-up visits at university hospitals, collaborating clinics or private physicians' practices [8], [9]. The protocol was approved by all local ethical committees and all patients gave written informed consent.

### Statistical Methods (If Applicable)

We decided to perform separate analyses for patients who initiated treatment in the mono/dual drug combination era and for patients who started with cART because of the potential survivor bias in the former group. Furthermore, preliminary analyses showed pronounced interactions between era of starting ART and the impact of previously failed regimens as well as the impact of suboptimal adherence. First, we performed descriptive analyses of the proportion of patients with HIV-1 RNA below and above 50 copies/ml and with CD4 cell counts below and above 200/µl at visit 1, and analyzed the association between these markers and the number of previous virologically failed regimens. Second, we determined the virological status at visit 2, and the proportion of patients with treatment discontinuation, treatment modification or clinical progression to AIDS or death between visit 1 and 2. Third, we used uni- and multivariable logistic regression to analyze predictors for virological failure at visit 2. Covariables in these models included gender, age (grouped into <40, 40–44, 45–49 and 50+ years), non-white ethnicity, mode of HIV transmission, HCV co-infections, HIV-1 RNA (maximum ever, and ever <50 copies/ml prior to and at visit1), CD4 cell counts (nadir and at visit 1), adherence to therapy, total duration of antiretroviral therapy (5 year strata) and number of previous failed regimens. To assess whether the exclusion of patients starting with non-standard ART affected our conclusions, we performed a sensitivity analysis in which these patients were combined with patients from the cART era. We used Stata 10.0 (StataCorp, College Station, Texas, USA).

## Results

### Patient Selection

**Figure 1:**
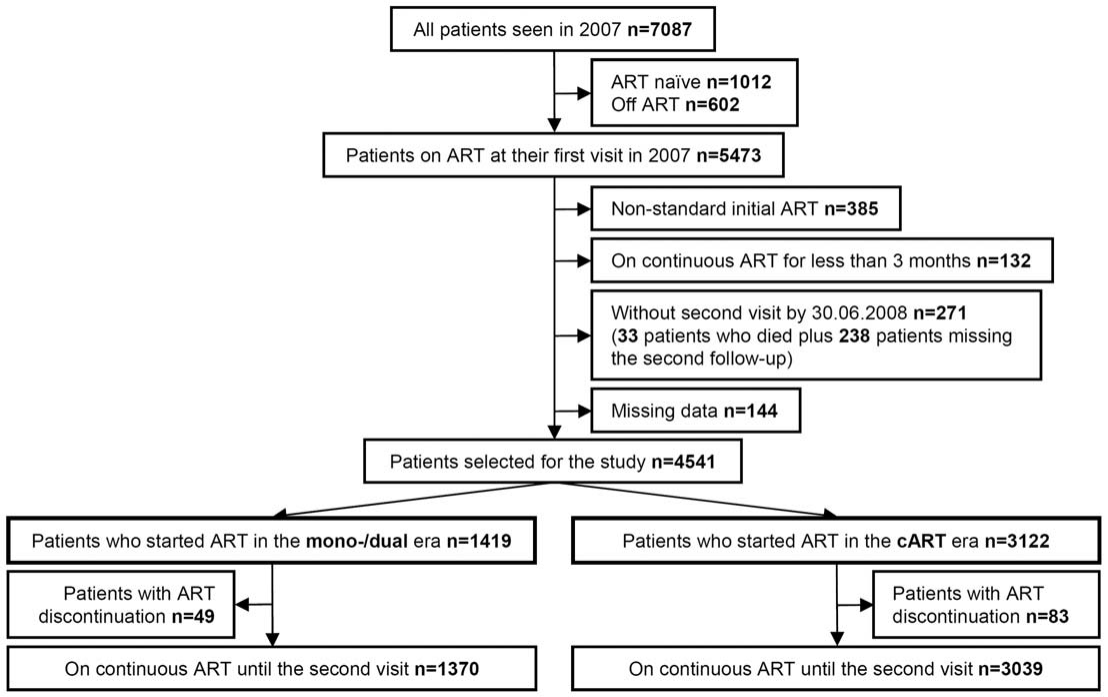
The patient disposition for this study is based upon all patients seen in the Swiss HIV Cohort Study during 2007.

The patient selection process is depicted in Figure 1. At their first visit in 2007. 5473 patients were on ART. Of these 385 were excluded because of non-standard initial drug regimens when starting ART between 1995 and 1997, the years of transition from mono/dual therapy to cART. 342 regimens were with single PI or single NNRTI plus single NRTI and 43 with other non-standard regimens. In addition, 547 patients were excluded due to various reasons. Included and excluded patients were similar with regards to gender, transmission risk group, CDC stage C at visit 1 (all p>0.5). However, excluded patients were on average 1 year younger (45 vs. 46 years, P = 0.003). The present analysis is thus based on 4541 patients of whom 1419 (31.2%) initiated ART with mono/dual therapies and 3122 (68.8%) with cART.

### Patient Characteristics at Visit 1 (Table 1)

**Figure 2:**
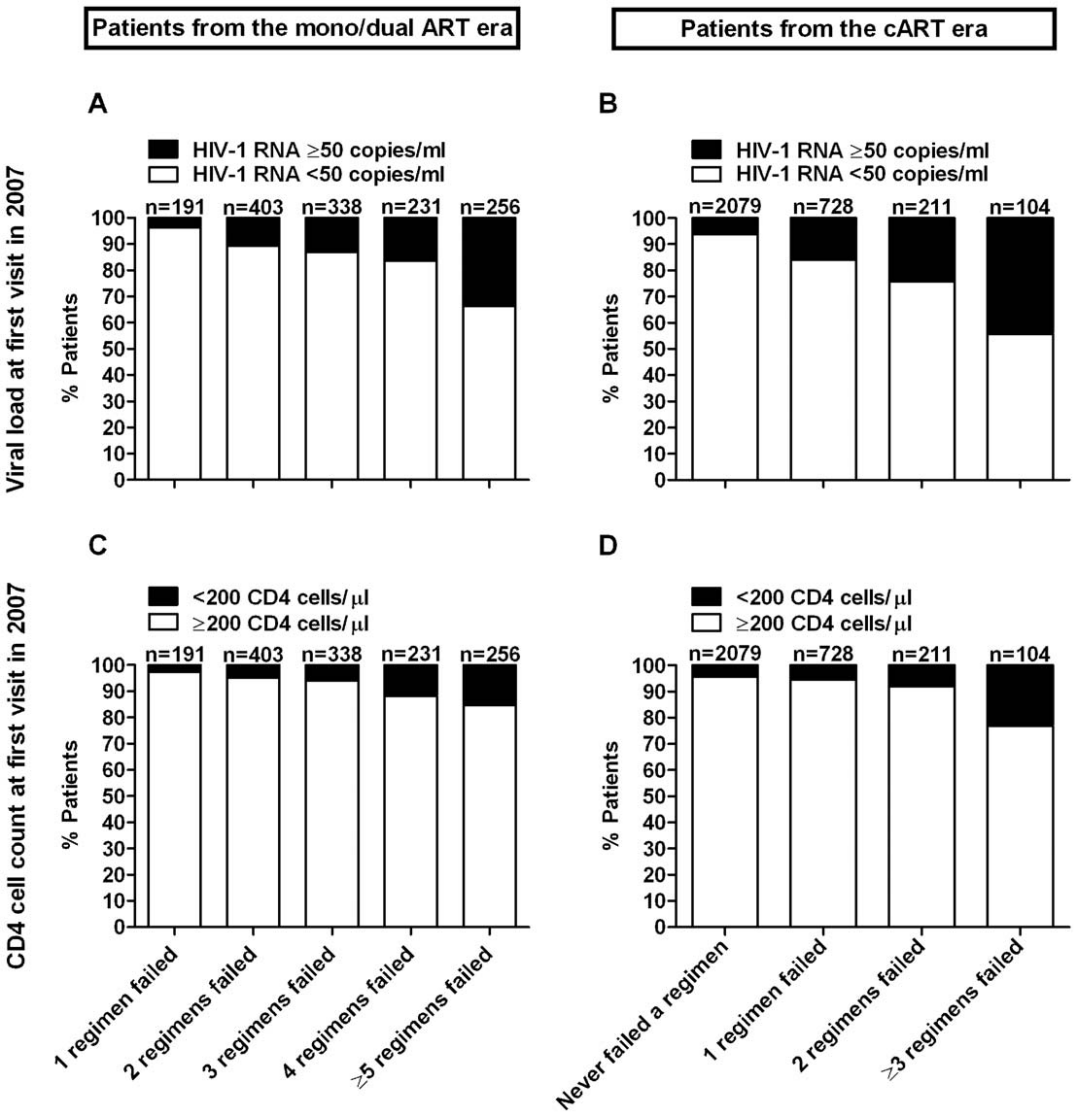
Distribution of viral loads and CD4 cell counts at first follow-up visit in 2007 (visit 1) according to the number of previous virologically failed regimens. The upper panels show HIV-1 RNA counts for patients who started therapy in the mono/dual era (A) and in the cART era (B). Lower panels show CD4 cell counts for patients from the mono/dual era (C) and for patients from the cART era (D), respectively. By definition all patients from the mono/dual era failed at least one regimen.

Reflecting the changing epidemiology of HIV in Switzerland, patients who started with mono/dual treatments were more frequently of white ethnicity, infected via needle sharing and thus with active HCV co-infection, had higher CD4 cell counts at enrolment in the SHCS, but lower nadir CD4 cell counts thereafter. At visit 1, these patients had more advanced HIV disease (74.3% vs. 52.6% in clinical CDC stages B or C), had experienced more virologically failed regimens in the past (average 3.2 vs. 0.5), and the proportion with HIV-1 RNA <50 copies/ml was lower (84.6% vs. 89.1%). Irrespective of the era of treatment initiation, higher numbers of previous failed regimens were strongly associated with higher proportions of detectable HIV-1 RNA at visit 1 (chi-square test for linear trend, p<0.001) and of CD4 cell counts <200 cells/µl (p<0.001) at visit 1 (Figure 2).

**Table 1:** Characteristics at the first semiannual follow-up visit in 2007 (visit 1) comparing individuals who started with mono/dual ART and cART.

Category	Subcategory	mono/dual ART	cART	p-value^1^
Number of patients (%)		1419 (31.3)	3122 (68.7)	
Sex (%)	Female	398 (28.1)	947 (30.3)	0.118
Age – median years (IQR)		47 (43–53)	44 (38–50)	<0.001
Ethnicity (%)	White	1281 (90.3)	2492 (79.8)	<0.001
	Other	138 (9.7)	630 (20.2)	
Transmission category (%)	Heterosexual	402 (28.3)	1351 (43.3)	<0.001
	Injecting Drug Use	393 (27.7)	464 (14.8)	
	Homosexual	571 (40.2)	1164 (37.3)	
	Other	53 (3.8)	143 (4.6)	
Active hepatitis B co-infection (%)		89 (6.3)	171 (5.5)	0.285
Active hepatitis C co-infection (%)		356 (25.1)	459 (14.7)	<0.001
CD4 at cohort inclusion – median cells/µl (IQR)		340 (185–520)	304 (162–488)	<0.001
Nadir CD4 cell count – median cells/µl (IQR)		120 (48–204)	172 (78–257)	<0.001
Max. HIV-1 RNA - median log_10_ copies/ml (IQR)		5.0 (4.4–5.5)	5.1 (4.6–5.6)	0.004
Ever had undetectable viral load (%)		1374 (96.8)	3042 (97.4)	0.245
Clinical CDC Stage (%)	A	365 (25.7)	1480 (47.4)	<0.001
	B	597 (42.1)	805 (25.8)	
	C	457 (32.2)	837 (26.8)	
CD4 cell count – median cells/µl (IQR)		492 (353–696)	496 (353–680)	0.981
HIV-1 RNA <50 (%)		1201 (84.6)	2781 (89.1)	<0.001
ART regimen (%)	3 NRTI only	63 (4.4)	309 (9.9)	<0.001
	Unboosted PI based	108 (7.6)	187 (6.0)	
	Boosted PI based	554 (39.0)	1174 (37.6)	
	NNRTI based	344 (24.2)	1279 (41.0)	
	3 class regimen	215 (15.2)	93 (3.0)	
	Any drug + T-20	59 (4.2)	13 (0.4)	
	Other	76 (5.4)	67 (2.1)	
Total ART duration (years) – median years (IQR)	11.7 (10.9–13.8)	5.7 (2.8–8.7)	<0.001	
Adherence (in the past 4 weeks, %)	Never missed a dose	1158 (81.6)	2574 (82.5)	0.024
	Missed 1 dose	152 (10.7)	376 (12.0)	
	Missed 2 doses	54 (3.8)	79 (2.5)	
	Missed >2 doses	55 (3.9)	93 (3.0)	
Number of ART regimens previously failed (%)	0	- 2	2079 (66.6)	<0.001
	1	191 (13.5)	728 (23.3)	
	2	403 (28.4)	211 (6.8)	
	3	338 (23.8)	63 (2.0)	
	4	231 (16.3)	22 (0.7)	
	≥5	256 (18.0)	19 (0.6)	
	mean – (range)	3.2 (1–14)	0.5 (0–8)	<0.001

1 P-values for comparison of mono/dual and cART era are calculated from chi-square tests (categorical variables) or from Wilcoxon-Mann-Whitney tests (continuous variables).

2 By definition, one failure event was added to all patients from the mono/dual era. Therefore, no patients from the mono/dual era can have 0 regimen previously failed.

### Virological Outcome at Visit 2

**Figure 3:**
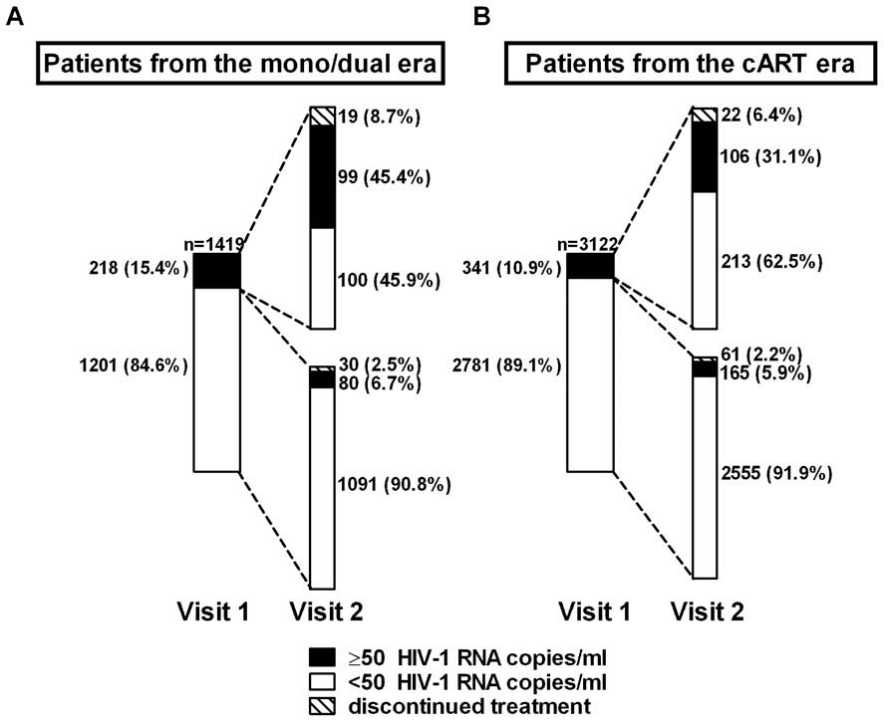
Virological course between the first follow-up visit in 2007 (visit 1) and the next semi-annual follow-up cohort visit (visit 2). Viral loads patterns are shown in the upper panels: (A) patients from the mono/dual ART era; (B) patients from the cART era.

Figure 3 depicts the virological course between the two visits. The median time between the visits was 6.3 months (IQR: 5.8–7.2). Of the patients with mono/dual treatment initiation, 15.4% had a detectable viral load at visit 1. Among these, complete suppression of viral replication was reached in 45.9% at visit 2. Fewer patients (10.9%) who started with cART had a detectable viral load at visit 1, and a higher percentage (62.5%) attained a viral load <50 copies/ml at visit 2 (p<0.001). On the other hand, of those with undetectable viral load at visit 1, 6.7% in the mono/dual vs. 5.9% in the cART group had a virologic failure with HIV-1 RNA ≥50 copies/ml at visit 2 (P = 0.37).

**Figure 4:**
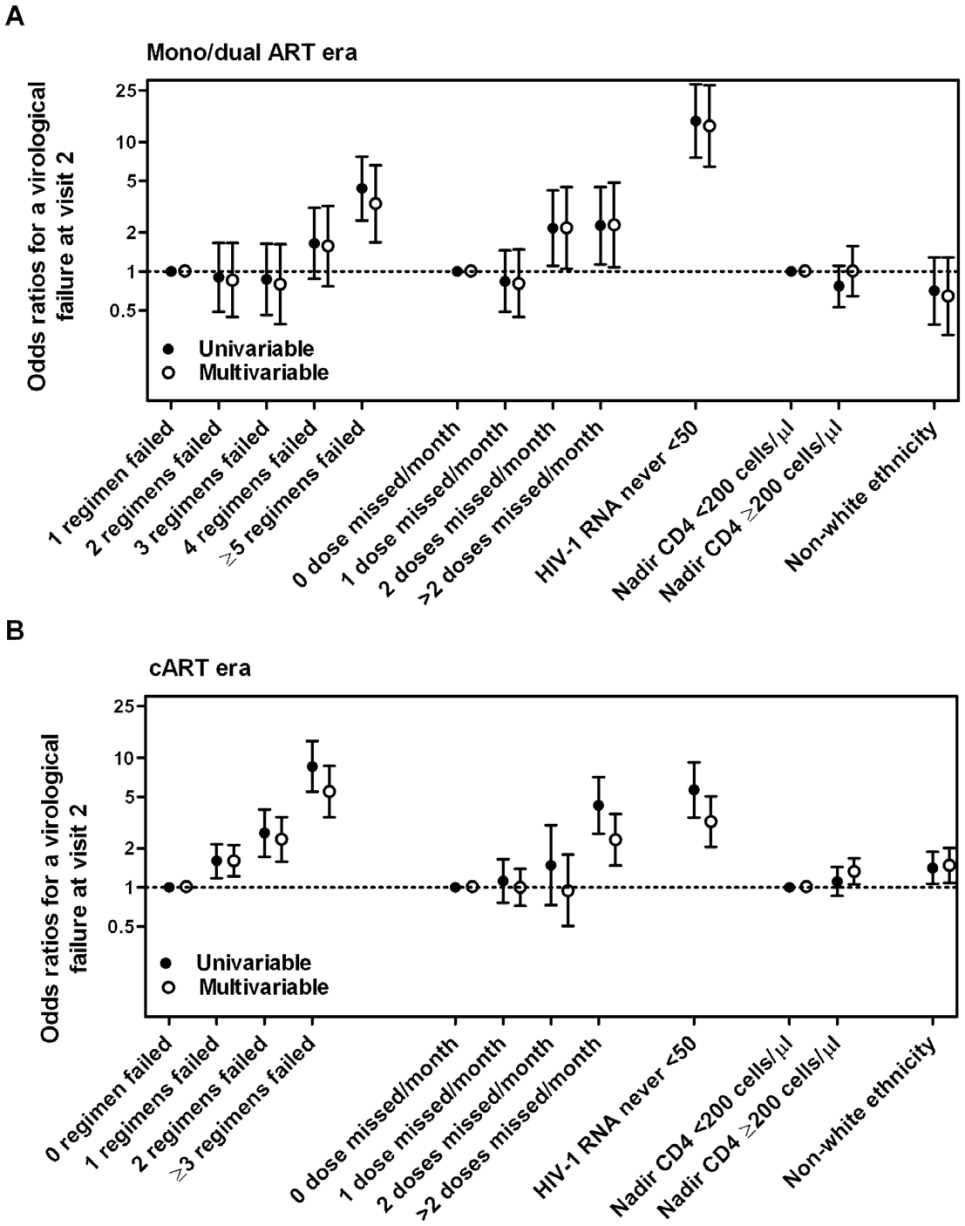
Factors associated with HIV-1 RNA >50 copies/ml at visit 2. Odds ratios from uni- and multivariable logistic regressions are shown with 95% confidence intervals. Panel A includes patients who initiated ART the mono/dual era, and panel B those who initiated ART in the cART era. Multivariable models were also adjusted for age, sex, transmission risk group, HCV co-infection, total duration of ART therapy, maximum viral load ever and CD4 cell count at the first visit in 2007 (all p-values >0.05).

Among all patients without treatment discontinuations between the two visits, 179/1370 (13.1%) from the mono/dual and 271/3039 (8.9%) from the cART era had HIV-1 RNA ≥50 copies/ml at visit 2 (p<0.001). Results from uni- and multivariable logistic regression analyses for both eras are shown in Figure 4. Virological failures were independently associated with the number of previous failed regimens, poor adherence, and lack of having ever reached complete viral suppression. Adjusted estimates for the number of previously failed regimen modeled as continuous variables indicated a steeper association with virological failure in patients from the cART era compared to the mono/dual era: odds ratio per previous regimen failed of 1.87 (95% CI 1.58–2.21) vs. 1.49 (1.28–1.73). In addition, individuals of non-white ethnicity from the cART era are more likely to have detectable viral load at visit 2: adjusted odds ratio of 1.59 (1.09–2.31). This can be partly explained by lower adherence levels among patients of non-white ethnicity: perfect adherence was reported by 79.7% of non-white vs. 82.7% (test for trend across adherence categories: P = 0.031).

The number of previously failed regimens remained a significant predictor of virological outcome at visit 2 when limiting the analysis to patients who had undetectable HIV-1 RNA levels at visit 1 with Odds Ratios of 2.33 (1.18–4.62) for ≥5 previously failed regimens among patients who started in the mono/dual era and 5.13 (2.62–10.0) for ≥3 previously failed regimens among patients who started in the cART era.

Results from a sensitivity analysis in which we included patients who started with non-standard ART to the cART group were virtually identical.

### Treatment Discontinuations and Modifications

During the six months separating the two visits, 49/1419 (3.5%) patients from the mono/dual era and 83/3122 (2.7%) patients from the cART era discontinued treatment for 15 days or longer (P = 0.14). Reasons for discontinuation were patient's wish (69.4% for patients from the mono/dual vs. 62.7% for patients from the cART era) or physician's decision (16.3% vs. 8.4%), drug toxicity (4.1% vs. 8.4%), and others (10.2% vs. 20.5%). Treatment modifications over the two consecutive visits were observed among 358/1419 (25.2%) patients from the mono/dual era and 768/3122 (24.6%) from the cART era (P = 0.65). 23/1419 (1.6%) patients from the mono/dual era intensified treatment by adding a drug; the other 335 (23.6%) stopped one or more drugs without completely discontinuing treatment. Similarly, 47/3122 (1.5%) patients from the cART era intensified treatment and 721/3122 (23.1%) stopped taking at least one of the drugs. Reported reasons for stopping a drug were virological, immunological, or clinical failure (9.0% patients from the mono/dual vs. 4.7% from the cART era), metabolic disorders (6.6% vs. 7.1%), gastro-intestinal disorders (2.4% vs. 2.8%), other toxicities (9.9% vs. 8.7%), or other reasons such as patient's wish or physician's decision (72.1% vs. 76.7%).

### Clinical Course

New clinical AIDS events between visit 1 and visit 2 occurred in 1 patient from the mono/dual era (non-Hodgkin lymphoma) and 4 patients from the cART era (3 non-Hodgkin lymphoma, 1 extrapulmonary tuberculosis). Not included in the above analyses are the 33 patients who died before visit 2 (Figure 1). Of these, 15 had initiated treatment in the mono/dual era and 18 in the cART era. For 4 patients the primary cause of death was attributed to HIV, 2 committed suicide, 1 died of an overdose of narcotics and for 5 patients the causes of death were unknown. The causes of death for the remaining 21 patients based upon ICD-10 codes were: 5 liver failures, 2 liver carcinoma, 1 gastrointestinal haemorrhage, 2 acute peritonitis, 4 lung cancers, 1 breast cancer, 2 pneumonia, 2 septicemia and 2 cardiovascular events.

## Discussion

We studied the impact of patients' treatment history, previous virological failures and adherence at two semiannual visits in 2007/08 among a cohort of 4541 participants on stable cART. At visit 1 the percentage of patients with viral loads <50 copies/ml was high with 84.6% of patients who started ART in the mono or dual therapy era and 89.1% among those who started with cART directly. Nevertheless, between 5 and 7% of these successfully treated patients experienced virological failure until visit 2 after six months. In the analysis of predictors for virological failure at visit 2 we found that the main independent risk factors were the number of previous failed regimens, suboptimal adherence to therapy and never having achieved an undetectable viral load.

We observed that approximately 3% of patients discontinued treatment for 2 weeks or longer and 25% modified treatment between the two semiannual visits. A recent study investigating treatment switches and interruptions in the SHCS showed that changes are frequent: up to 48% of the patients change treatment within 12 months after treatment initiation. Intolerance is the main reason for treatment switches, whereas discontinuation is equally explained by both intolerance and patient's wish [10].

The proportion of 85–90% of treated patients in routine clinical care having undetectable viral loads is similar to what has been shown for randomized clinical trials of treatment naive patients [2]–[4]. In contrast to clinical trials which usually have stringent inclusion and exclusion criteria, patients in our study are largely unselected and representative. In fact, a comparison of drug sales data for Switzerland (Source: IMS Health GmbH, Sonnenbergstrasse 11, 6052 Hergiswil, Switzerland) with treatment data of the SHCS for 2007 showed that 74% of the NRTI compounds sold in the country had been consumed by individuals followed in the SHCS.

Few long-term studies have directly compared patients from the mono/dual treatment and the cART era. The distinct group of patients, who started treatment already in the pre-cART era and survived, has now been on cART for more than 10 years. Most of these patients were only on partially suppressive treatments before cART [11], which influenced the course of their HIV infection [12], [13]. Many of them also experienced virological failure on cART regimens taken thereafter. Archived resistance mutations can lead to treatment failure of subsequent regimens [14], and therefore, further treatment options are compromised. In 1999, three years after cART was routinely available, we observed a worse virological outcome among patients who had been pre-treated with mono/dual therapies with >35% viral rebounds, vs. 20% for those who initiated therapy with cART [9]. In the EuroSida study, six years after treatment initiation, up to 20% of the patients experienced multiple drug class failure which was associated with poorer clinical status [15]. A more recent multi-cohort analysis showed that the risk for virological failure was reduced by at least 50 percent between 1996 and 2002 among treatment-naïve persons starting cART [16].

The association between the history of treatment failures and later viral break-through has been shown in previous studies [6], [17]. Prior virologic failure doubled the risk of subsequent virologic failure in a cross-sectional study [17], and viral rebound rates were associated with the number of regimens previously failed, the risk increasing by 38% for each failed regimen [6]. It is likely that HIV-1 resistance mutations had been accumulated in such patients with repetitive virological failures but a resistance test at visit 1 would not have been feasible in the vast majority of patients due to suppressed viral replication.

Although the assessment of adherence is not uniform and subject to methodological bias, the relationship between poor adherence and virological failure is not disputed [7], [18], [19]. The SHCS documents self-reported adherence within the previous month, which was found to reliably correlate with viral rebounds [7], [20]. Adherence may differ between regimens and once-daily regimens may be less forgiving. However, due to small numbers of patients on once-daily treatments further analyses were not possible in the present study. We cannot fully explain the impact of ethnicity on virological failure in our study because migrants have unrestricted access to care and medication in our country [21]. However, we assume that socio-economic status which is often lower in migrant population in Switzerland, as well as psychosocial and language barriers negatively affect adherence and treatment outcomes [18], as has been observed in other European cohorts [6], [22].

### Limitations

The design of the present study implied that patients had to survive until 2007, therefore selecting patients with good prognostic markers who initiated ART in the era of mono/dual therapies. In fact, 2688/5769 (47%) patients who initiated ART with a mono/dual regimen in the SHCS died before 2007, vs. only 349/5191 (6.7%) patients who started therapy with cART. Our study reflects the current situation of the present heterogeneous patient population in a country with universal access to care. Thus, extrapolations to other settings, especially in developing countries need to be done with caution. An additional limitation by design is the short observation period of 6 months which precludes the analysis of events that require a longer time to occur. On the other hand, this relatively short follow-up period was deemed to represent the typical clinical situation of routine patient care.

### Conclusions

Although antiretroviral treatment is very successful, lack of continued viral suppression on stable cART is still relatively frequent in today's practice. Major information on risks for virological failure is the accurate patient's history, including the history of mono/dual drug therapy, the number of previous regimens with virological failure, and adherence to therapy. The former factors are associated with archived resistance mutations and mandate a careful selection of drugs in case of a treatment change, even if the current resistance testing - if at all possible - may not reveal all accumulated resistance mutations. Maintenance of good adherence to therapy is key of patient care and long-term suppression of viral replication, especially with the promising new drugs and drug classes currently entering routine care.
